# Inequalities in COVID-19 mortality: defining a global research agenda

**DOI:** 10.2471/BLT.22.288211

**Published:** 2022-09-02

**Authors:** Joseph Friedman, Mirza Balaj, Nazrul Islam, Youyang Gu, Petra Nahmia, Carolina Santamaría-Ulloa, Andres Gutierrez Rojas, Kumanan Rasanathan, Ahmad Reza Hosseinpoor, Jacques BO Emina, Terje Andreas Eikemo, Carlos Castillo-Salgado

**Affiliations:** a Center for Social Medicine and Humanities, University of California, B7-435, UCLA Semel Institute, Los Angeles, CA 90095-1759 United States of America (USA).; b Centre for Global Health Inequalities Research, Norwegian University for Science and Technology, Trondheim, Norway.; c Nuffield Department of Population Health, University of Oxford, Oxford, England.; d New York, USA.; e Statistics Division, United Nations Economic and Social Commission for Asia and the Pacific, Bangkok, Thailand.; f Institute for Health Research, University of Costa Rica, San Jose, Costa Rica.; g Statistics Division, United Nations Economic Commission for Latin America and the Caribbean, Santiago, Chile.; h Department of Social Determinants of Health, World Health Organization, Geneva, Switzerland.; i Department of Data and Analytics, World Health Organization, Geneva, Switzerland.; j Department of Population and Development Studies, University of Kinshasa, Kinshasa, Democratic Republic of the Congo.; k Bloomberg School of Public Health, Johns Hopkins University, Baltimore, USA.

The global death toll of the coronavirus disease 2019 (COVID-19) pandemic is very high, with over 6 million officially registered deaths and estimates of excess mortality ranging from 10 million to 20 million.^1^^–^^3^ Yet this burden has not been equally distributed between countries or across race, ethnicity, socioeconomic status and social class within countries.^4^^–^^6^ Evidence from several countries indicate disparities in exposure, susceptibility and capacity to treat and contain infection, severe illness, hospitalization and death stemming from the disease.^7^ Leading scholars have described COVID-19 as a syndemic (that is, where social and biological factors interact to produce poor health outcomes), as mortality and morbidity from the pandemic feed into and exacerbate existing inequalities in social conditions and chronic disease rates.^4^^–^^6^ An early systematic review revealed stark social inequalities in mortality in the early months of the pandemic among a subset of high-income countries.^7^ A recent World Health Organization (WHO) evidence brief identified evidence of poorer COVID-19-related outcomes within countries for lower income individuals, marginalized ethnic minorities, indigenous people, low-paid essential workers, migrants, populations affected by emergencies (including conflicts), incarcerated populations and people experiencing homelessness and housing insecurity.^8^

Although evidence suggests that the pandemic has exacerbated social inequalities in mortality, a global synthesis of the trajectory of COVID-19 is needed. Furthermore, quantitative data synthesis is required to understand the global magnitude of inequalities in COVID-19 mortality, as measured with respect to a diverse set of social stratifiers (that is, measures of socioeconomic position, such as educational attainment or wealth). We also need more clarity to ascertain the global picture of the theoretical and methodological approaches underpinning COVID-19 mortality inequality research.^4^

The Technical Advisory Group on COVID-19 Mortality Assessment advises and supports efforts by WHO and the United Nations Department of Economic and Social Affairs on matters related to COVID-19 mortality. Working Group 5, on inequality in COVID-19 mortality between and within countries, provides evidence-based recommendations regarding the study of demographic, socioeconomic and geographical inequalities in COVID-19 mortality.^9^

Here, we detail the global research agenda defined by this working group to assess the state of existing scientific knowledge regarding social inequalities in COVID-19 mortality, synthesize research about the scope and magnitude of inequalities, and identify key gaps for ongoing data collection and study. A team of researchers housed at the Centre for Global Health Inequalities Research at the Norwegian University for Science and Technology in Trondheim is undertaking this work along with the leadership of the Global Public Health Observatory of the Johns Hopkins Bloomberg School of Public Health,^10^ under supervision of the Inequality Working Group within the Technical Advisory Group^9^ and in collaboration with a global network of researchers. We suggest that a two-phase, systematic assessment is well suited to address the research questions. The first phase will be aimed at determining the existing frameworks and data coverage describing social inequalities in COVID-19 mortality, and which social stratifiers these frameworks have focused on. The second phase will be focused on quantitatively synthesizing the effect sizes of a key set of social stratifiers for COVID-19 mortality.

The first phase consists of charting the landscape of frameworks and stratifiers that have been used to measure COVID-19 inequalities. A systematic search of the literature will be carried out leveraging several databases such as: PubMed®, Web of Science, Scopus, Embase®, Global Health, EconLit and Sociology Source Ultimate. The search will be limited to papers published on the subject of review since February 2020 without any restrictions on language, sample size or characteristics. The phenomenon of interest is adult COVID-19 mortality based on social position, broadly defined using a wide range of social markers, including educational attainment, household wealth, income, race, ethnicity, urbanicity, employment/occupational status and insurance status, as available. Both individual and area-level measures will be assessed in this phase. Age and sex will be assessed where they are studied intersectionally with other social dimensions, such as income or education. A set of pilot searches identifying key papers on social inequalities in adult COVID-19 mortality will guide the development of a list of social stratifiers and theoretical frameworks. Theoretical frameworks will likely include the syndemic approach, intersectionality, fundamental cause theory, social determinants and straightforward social epidemiological measurement approaches. Study designs for represented research will include cohort studies, cross-sectional studies, randomized controlled trials and non-randomized trials. Extracted quantitative measures of effect size will include relative risk, hazard ratio, odds ratio and rate ratio as they describe official direct COVID-19 mortality as well as excess mortality. Preprints and other doi (digital object identifier)-referenced articles will be included; however, viewpoint pieces will be excluded.

Two researchers – with a third in case of discrepancy – will screen all titles and abstracts of identified references. Researchers working in pairs and applying the inclusion and exclusion criteria identified will also perform full-text reading. After the selection of included studies, the information regarding month(s) and year(s) of data assessed, country, population and age group, study design and method used, risk estimate, confidence intervals and sample size will be extracted out of each study and included in a database. Two qualitative review rounds will take place, one mid-term review and one at the end of the extraction phase.

The first phase will conclude with a summary of the social stratifiers, geographical coverage and theoretical frameworks employed in the existing corpus of work. Leveraging the extracted database, we will quantify the geographical coverage of work describing inequalities in COVID-19 mortality. Given limitations in data infrastructure, we expect to find a preponderance of studies for high-income countries, which would represent a critical gap that should be improved in research moving forward. We will also be able to describe the social stratifiers that have been assessed for each world region. Finally, we can show which kinds of inequality metrics, and which frameworks have been employed worldwide. This first stage assessment aims to serve as a guide for ongoing research on inequalities, to describe the existing state of knowledge and identify key gaps as well as strengths in the current corpus of studies.

The second phase will quantify the global magnitude of inequalities in COVID-19 mortality. In this phase, we propose to quantitatively synthesize results describing inequalities in COVID-19 mortality globally for a key set of social stratifiers. The final designation regarding the choice of stratifiers will be made once the database has been established, allowing for the assessment of the most represented indicators. However, we expect educational attainment, income, wealth, and employment type and status to serve as key indices. In this phase we propose to focus on individual-level measures, not area-level (such as postal code or municipality) measures, to improve comparability and standardization of measures.

In line with previous meta-regression analyses published by researchers involved in this endeavour^11^ we will leverage the Meta Regression – Bayesian Trimmed Regularized framework, which was developed as part of comparative risk assessment work conducted for the Global Burden of Disease Study.^12^ Using mixed-effect meta-regression, we will combine all measures of the relationship between COVID-19 mortality and a given social stratifier, adjusting for study design, the inclusion of study-level confounders and covariates, the uncertainty associated with each point estimate of measured effect and heterogeneity between studies. Consistent with prior applications of this approach,^11^ cross-walking will be used to standardize differences in effect size based on outcome measure type, for example between direct COVID-19 mortality and excess mortality. This method is critical as direct COVID-19 mortality estimates are known to underestimate total pandemic-related deaths, with a social gradient in undercounting and out-of-hospital death.

## An invitation to collaboration 

This study of global health inequalities must be conducted as a global, collaborative endeavour to be successful. A key aspect of this research will entail the development of a COVID-19 Mortality Inequality Collaborator Network, consisting of interested researchers with relevant expertise from diverse world regions and academic backgrounds. Collaborators will participate in identifying and addressing data gaps, reviewing model analyses, guiding the interpretation of findings and developing peer-reviewed articles. We encourage interested candidates, especially those from underrepresented backgrounds and from low- and middle-income countries, to join this project’s collaborator network. A doctoral or master’s degree, or equivalent experience, and expertise in the measurement of social inequalities in mortality in country of origin or professional context are desired characteristics of collaborator network candidates. At a minimum, collaborator network members can expect to provide structured feedback at several stages of the research process, including reviewing identified data sources, analytical outputs and manuscript drafts. More information can be found at the working group’s website.^9^

Although this work will be initially limited to studying inequalities in mortality stemming from the COVID-19 pandemic, the study will lay the groundwork for subsequent research regarding inequalities in morbidity, which is especially relevant in light of the long-term sequelae experienced by many COVID-19 patients. We expect this research endeavour will result in a comprehensive summary of research describing inequalities in COVID-19 mortality, summarizing the status of current research in the field, and identifying key gaps for future efforts in this area. These results will be invaluable to the Inequality Working Group in making recommendations regarding key priority areas moving forward, as well as opportunities to coordinate data generation and analyses between countries.

## References

[1] COVID-19 map [internet]. Baltimore: Johns Hopkins Coronavirus Resource Center; 2022. Available from: https://coronavirus.jhu.edu/map.html [cited 2022 August 24].

[2] Karlinsky A, Kobak D. Tracking excess mortality across countries during the COVID-19 pandemic with the World Mortality Dataset. eLife. 2021 Jun 30;10:e69336. 10.7554/eLife.69336 34190045 PMC8331176

[3] Wang H, Paulson KR, Pease SA, Watson S, Comfort H, Zheng P, et al. Estimating excess mortality due to the COVID-19 pandemic: a systematic analysis of COVID-19-related mortality, 2020–21. Lancet. 2022;399(10334):1513–36. 10.1016/S0140-6736(21)02796-3 35279232 PMC8912932

[4] Bambra C. The unequal pandemic: COVID-19 and health inequalities. 1st ed. Bristol: Policy Press; 2021.

[5] Islam N, Lacey B, Shabnam S, Erzurumluoglu AM, Dambha-Miller H, Chowell G, et al. Social inequality and the syndemic of chronic disease and COVID-19: county-level analysis in the USA. J Epidemiol Community Health. 2021 Jan 5;75(6):496–500. 10.1136/jech-2020-215626 33402397

[6] Horton R. Offline: COVID-19 is not a pandemic. Lancet. 2020 Sep 26;396(10255):874. 10.1016/S0140-6736(20)32000-6 32979964 PMC7515561

[7] Wachtler B, Michalski N, Nowossadeck E, Diercke M, Wahrendorf M, Santos-Hövener C, et al. Socioeconomic inequalities and COVID-19 - a review of the current international literature. J Health Monit. 2020 Oct 9;5 Suppl 7:3–17. 35146298 10.25646/7059 PMC8734114

[8] COVID-19 and the social determinants of health and health equity: evidence brief. Geneva: World Health Organization; 2021. Available from: https://apps.who.int/iris/handle/10665/348333 [cited 2022 Feb 20].

[9] Inequality in COVID-19 mortality between and within countries [internet]. Geneva: World Health Organization; 2022. Available from: https://www.who.int/data/technical-advisory-group/covid-19--mortality-assessment/working-group-5 [cited 2022 Aug 30].

[10] Castillo-Salgado C. Developing an academia-based public health observatory: the new global public health observatory with emphasis on urban health at Johns Hopkins Bloomberg School of Public Health. Cad Saude Publica. 2015 Nov;31 Suppl 1:286–93. 10.1590/0102-311X00132914 26648383

[11] Balaj M, York HW, Sripada K, Besnier E, Vonen HD, Aravkin A, et al. Parental education and inequalities in child mortality: a global systematic review and meta-analysis. Lancet. 2021 Aug 14;398(10300):608–20. 10.1016/S0140-6736(21)00534-1 34119000 PMC8363948

[12] Murray CJL, Aravkin AY, Zheng P, Abbafati C, Abbas KM, Abbasi-Kangevari M, et al.; GBD 2019 Risk Factors Collaborators. Global burden of 87 risk factors in 204 countries and territories, 1990-2019: a systematic analysis for the Global Burden of Disease Study 2019. Lancet. 2020 Oct 17;396(10258):1223–49. 10.1016/S0140-6736(20)30752-2 33069327 PMC7566194

